# Integrating subject-specific workspace constraint and performance-based control strategy in robot-assisted rehabilitation

**DOI:** 10.3389/fnins.2024.1473755

**Published:** 2024-10-30

**Authors:** Qing Miao, Song Min, Cui Wang, Yi-Feng Chen

**Affiliations:** ^1^School of Electrical and Electronic Engineering, Wuhan Polytechnic University, Wuhan, China; ^2^Shenzhen Key Laboratory of Smart Healthcare Engineering, Guangdong Provincial Key Laboratory of Advanced Biomaterials, Department of Biomedical Engineering, Southern University of Science and Technology, Shenzhen, China

**Keywords:** robot-assisted rehabilitation, integrated framework, compliant motion constraint, iterative learning, RBFNN control structure

## Abstract

**Introduction:**

The robot-assistive technique has been widely developed in the field of neurorehabilitation for enhancement of neuroplasticity, muscle activity, and training positivity. To improve the reliability and feasibility in this patient–robot interactive context, motion constraint methods and adaptive assistance strategies have been developed to guarantee the movement safety and promote the training effectiveness based on the user’s movement information. Unfortunately, few works focus on customizing quantitative and appropriate workspace for each subject in passive/active training mode, and how to provide the precise assistance by considering movement constraints to improve human active participation should be further delved as well.

**Methods:**

This study proposes an integrated framework for robot-assisted upper-limb training. A human kinematic upper-limb model is built to achieve a quantitative human–robot interactive workspace, and an iterative learning-based repulsive force field is developed to balance the compliant degrees of movement freedom and constraint. On this basis, a radial basis function neural network (RBFNN)-based control structure is further explored to obtain appropriate robotic assistance. The proposed strategy was preliminarily validated for bilateral upper-limb training with an end-effector-based robotic system.

**Results:**

Experiments on healthy subjects are enrolled to validate the safety and feasibility of the proposed framework. The results show that the framework is capable of providing personalized movement workspace to guarantee safe and natural motion, and the RBFNN-based control structure can rapidly converge to the appropriate robotic assistance for individuals to efficiently complete various training tasks.

**Discussion:**

The integrated framework has the potential to improve outcomes in personalized movement constraint and optimized robotic assistance. Future studies are necessary to involve clinical application with a larger sample size of patients.

## 1 Introduction

A large majority of patients with injuries to the nervous system suffer from motor disability of limbs, which gravely affects the quality of life. Exploring effective treatments, particularly rehabilitation strategies, is one of the challenging goals in medicine (Moore et al., 2020; Wright et al., 2020; Wingfield et al., 2022; Shi et al., 2024). In neurorehabilitation, bilateral upper-limb training is an effective adjunct treatment that has shown positive promise for neuroplasticity as it induces the remodeling of premotor cortex (Luft et al., 2004; Cauraugh and Summers, 2005; Chen et al., 2010; Xie et al., 2022; Norris et al., 2024) and prepares human subjects to return to activities of daily living (Lim et al., 2016).

Traditional rehabilitation intervention is to build a one-to-one training environment by means of physical therapists. This way has been extensively adopted but not adequately improved owing to its low efficiency and precision (Zhang and Cheah, 2015). Based on such limitations, robot-assisted therapy has been recently developed by the controllability and repeatability. For industrial robots, accuracy, rapidity, and stability of the operation are recognized as the paramount importance (Koç et al., 2019; Han et al., 2020; Sun et al., 2023a; Sun et al., 2023b). However, there exists an additional subject who needs to operate the robotic device during rehabilitation training. In this scenario, information perception on the user’s movements becomes indispensable for a human–robot interaction, particularly in training safety and rehabilitation effectiveness.

When it comes to the human–robot interaction safety, a fundamental precondition is the estimation of suitable workspaces (Carbone et al., 2018). In general, some studies tend to drive the affected upper limb by referring the workspace of the unaffected side (Chunguang et al., 2009; Leonardis et al., 2015; Sarasola-Sanz et al., 2022), while the inconformity of both workspaces may result in strain injury. Other research studies prefer to try a standardized but small workspace according to the experience of the therapists (Squeri et al., 2009; Najafi et al., 2020). Although this one-size-fits-all approach can ensure the training safety by avoiding overstretch, the range of joint motion would not be sufficient, which may reduce the rehabilitation effectiveness. To address this problem, our previous study developed a subject-specific workspace determination method (Miao et al., 2020). The workspace was created based on a subject-specific upper-limb kinematic model. An attractive field was generated to guide the movement toward a predefined circle trajectory, and a repulsive field was defined to constrain deviated motion. Nevertheless, the diameter and position of the circle were set by the subjective opinion, which did not take into account individual adaptability. In addition, the variation of the resistance in the repulsive workspaces was uniformed rather than customized for the subjects. The inappropriate resistance levels may cause negative training, even “slacking” owing to the attractive field.

With regard to the training effectiveness, “assist-as-needed (AAN)” control techniques have been employed by providing only appropriate assistance during movement execution, which provides subjects more movement freedom. Pehlivan et al. (2016) proposed a minimal AAN controller for wrist rehabilitation robots in which the adaptive input estimation scheme included an extended Kalman filter with Lyapunov stability analysis. Zarrin et al. (2024) proposed a two-port admittance controller to address the lack of control frameworks for upper-limb rehabilitation exoskeletons. Cao et al. (2024) proposed a position-constrained AAN control method by introducing a constructed global continuous differentiable function incorporating dead zone and saturation characteristics to quantify the robotic assistance and facilitate seamless operation. It should be noted that the above-mentioned studies achieved good results; resorting to one-dimensional data, such as trajectory tracking error, movement velocity, or interactive force, is not comprehensive enough to support real estimation on subjects’ motor functions. This case would misguide device’s behavior of providing unsuitable assistance, which may cause patients’ negative emotions or intermittent slack during the training. Thus, it is important to evaluate the motion state accurately to formulate robotic subject-specific assistance for maximizing active participation of the patients.

To address this issue, performance-based control strategies have been proposed. These strategies are dependent on multiple kinematic indicators to comprehensively evaluate subjects’ motor functions, and adaptive controllers are designed to optimize robotic assistance based on the evaluation results. Krebs et al. detailed a concept of performance-based progressive robot therapy with MIT-MANUS, which included four diverse indicators in task-oriented training (Krebs et al., 2003). A piecewise function was adopted as an adaptive algorithm to tune the task difficulty. Similarly, Papaleo et al. presented a patient-tailored approach by using a seven degrees of freedom (DOFs) robot arm for three-dimensional (3D) upper-limb training (Papaleo et al., 2013). Three different performance indicators were developed to evaluate motor ability through a weighted sum method. Although these objective measures appear to be useful, they are not tightly linked to widely accepted clinical scales, such as the Fugl-Meyer Assessment (FMA), the Motor Status Score (MSS), or the modified Ashworth Scale, which may reduce the evaluation reliability of limbs’ motor ability. In addition, little attention was paid to the combination of the training safety and effectiveness, which affects the development of user-centered robotics.

This article contributes to the bilateral upper-limb rehabilitation by proposing an integrated framework for safe and feasible neurorehabilitation training. On the one hand, the framework introduces a subject-specific workspace design method based on human’s kinematic information at first; then, an iterative learning-based repulsive force field is established to perform optimal compliance motion constraints. On the other hand, a performance-based robotic assistance strategy is implemented to tailor subject-specific training task planning for various individuals. Three kinematic parameters of a clinical macro-metric model are applied as the performance indicators for accurate evaluation of subjects’ motor functions, and a radial basis function neural network (RBFNN)-based multi-objective optimization method is implemented to tailor training difficulty level.

The article is organized as follows: In Section 2, a detailed robot-assisted bilateral upper-limb training system is described, a safe interactive workspace with an iterative learning strategy is analyzed based on an end-effector robotic device, and an overall control architecture of the robotic device is described, including performance indicator acquisition and robotic assistance decision. Section 3 gives the experimental protocol and experimental results, and discussions and conclusion are included at the last section.

## 2 Methods

### 2.1 Robotic system configuration

**Figure 1:**
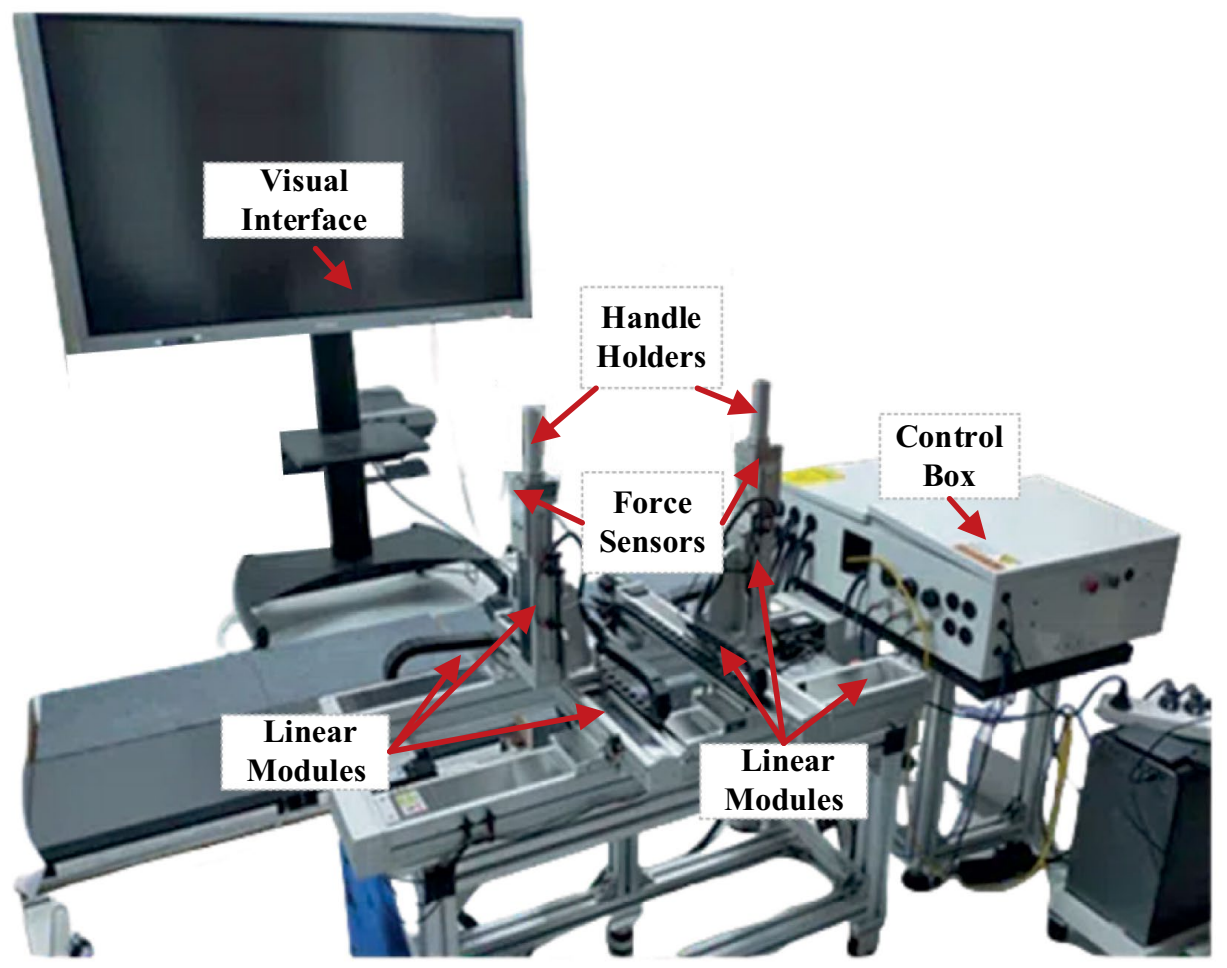
Bilateral upper-limb training system. It includes a visual interface, two handle holders, two three-axis force sensors, six linear modules, and a control box.

In this study, a robotic platform is applied for bilateral coordination training of human upper limbs, as shown in Figure 1 (Miao et al., 2021). The platform is a 6-DOF (two unilateral 3 DOF) end-effector device that comprises six linear modules and two handles with the aim of bilateral 3D movement. Each end-effector device is equipped with a three-axis force sensor to acquire interactive force data. In terms of software, the host computer of the system adopts LabVIEW developed by the NI company to set the control parameters, and it communicates with the lower controller CompactRIO in real time through an Ethernet cable. The communication mode between the servo system and CompactRIO is based on analog signal transmission. Then, the servo system provides position feedback to the controller in the form of a pulse signal via a digital acquisition module. The device is not only furnished with a stop button for emergency braking but also integrated with photoelectric switches for safety limits.

### 2.2 Workspace constraint construction

**Figure 2:**
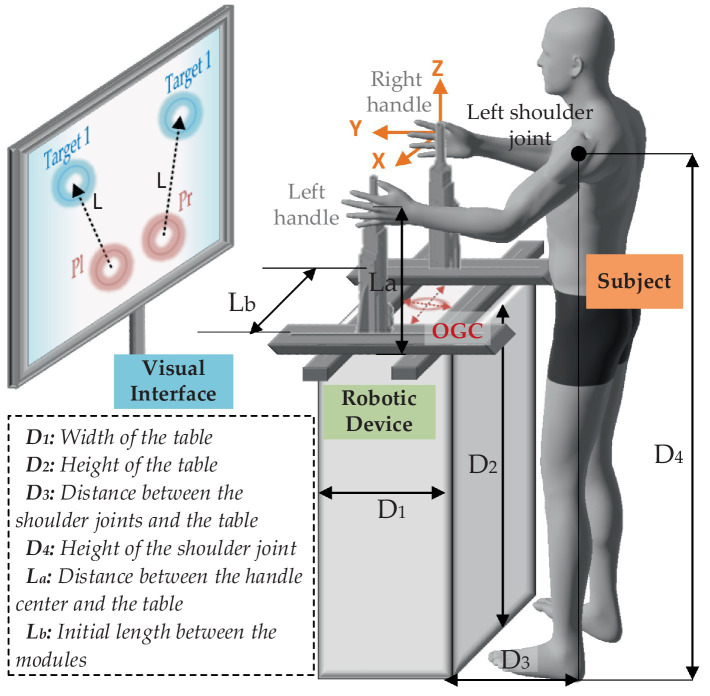
Schematic diagram of the proposed training strategy.

The mirror symmetry training has been widely used through the bilateral upper-limb rehabilitation, particularly useful for people suffering from hemiparesis. There are clear clinical findings that mirror training can improve therapy effectiveness against unilateral neglect. A schematic diagram of the bilateral training pattern is presented in Figure 2.

The global coordinate directions are described as the orange arrows, and the origin of global coordinate (OGC) is defined at the center of the four modules (as well as the center of the table). It is assumed that the dimensional positions of the robot and the subject are fixed. $D_{1}$ and $D_{2}$ indicate width and height of the table, respectively. During the training, the subject is asked to stand on the designated location (the center of the shoulder joints and OGC are on the same YZ plane), keep the body straight, grasp the handles, and focus on the training task presented on the visual interface. $D_{3}$ represents the distance between the shoulder joints and the table. $D_{4}$ is the height of the shoulder joint. $L_{a}$ is the distance between the handle center and the table. $L_{b}$ is the initial length between the modules on Y-axis. In this case, the reachable workspace of the handles can be obtained by coordinate transformation of the positions of the shoulders as given in Equations 1, 2.


(1) $$\left\{ \begin{array}{l} P_{\text{lx}}=PS_{\text{lx}}-S+0.5L_{b}\\ P_{ly}=PS_{ly}+0.5D_{1}+D_{3}\\ P_{lz}=PS_{lz}+L_{a}-D_{4} \end{array}\right.$$

(2) $$\left\{ \begin{array}{l} P_{rx}=PS_{rx}+S-0.5L_{b}\\ P_{ry}=PS_{ry}+0.5D_{1}+D_{3}\\ P_{rz}=PS_{rz}+L_{a}-D_{4} \end{array}\right.$$

where $PS_{l}=\left[PS_{\text{lx}},PS_{ly},PS_{lz}\right]$ denotes the coordinates of the left shoulder joint, and $PS_{r}=\left[PS_{rx},PS_{ry},PS_{rz}\right]$ denotes the coordinates of the right shoulder joint. S is the horizontal distance between each shoulder joint to the body center, which can be expressed as 0.179 times as the body height (Miao et al., 2018).

Therefore, the reachable interactive workspace can be described by quantitative upper-limb workspace. Our previous study proposed a three-stage method to determine human hands’ workspace on a subject-specific basis (Miao et al., 2018). This considered the human upper limb as a model with seven degrees of freedom and used the Denavit–Hartenberg (D-H) method to derive the human left-hand workspace $PS_{l}$ and the right-hand workspace $PS_{r}$ as given in Equation 3.


(3) $${\prod_{i=1}^{7}}^{i-1}A_{i}=\left[\begin{array}{cccc} n_{\text{lx}} & o_{\text{lx}} & \alpha_{\text{lx}} & PS_{x}\\ n_{ly} & o_{ly} & \alpha_{ly} & PS_{y}\\ n_{lz} & o_{lz} & \alpha_{lz} & PS_{z}\\ 0 & 0 & 0 & 1 \end{array}\right]$$

**Figure 3:**
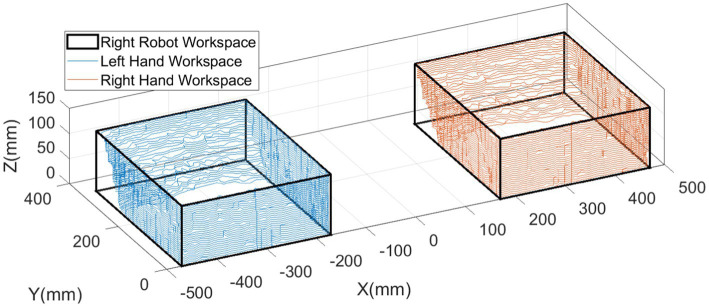
Workspaces of the handles.

(4) Ai−1Ai=cosθi−cosαisinθisinαisinθiaicosθisinθicosαicosθi−sinαicosθiaisinθi0sinαicosαidi0001

where $\alpha_{i}$, $a_{i}$, $d_{i}$, and $\theta_{i}$ are the D-H parameters of the ith upper-limb joint. Ai−1Ai is the homogeneous transformation matrix, as given in Equation 4. Then, the reachable interactive workspace can be plotted as shown in Figure 3.

To limit the movement into this workspace in safety, an optimized repulsive potential field concept is adopted to yield compliant constraint. Take the X-Y plane as an instance. It is assumed that the current position of the left handle is $P_{l}=\left(P_{\text{lx}},P_{ly}\right)$, and the right side is $P_{r}=\left(P_{rx},P_{ry}\right)$. To extend the line segment between handles and the geometric centers of the workspaces they located, we can obtain two points of intersections on the boundaries, and the ones positioning closer the boundaries are corresponding obstacles, denoted as $PO_{l}=\left(PO_{\text{lx}},PO_{ly}\right)$ for the left side and $PO_{r}=\left(PO_{rx},PO_{ry}\right)$ for the right side. The repulsive potential function can be presented as given in Equations 5, 6.


(5) $$U_{l}=\left\{\begin{array}{l} e^{-\eta \left[{\left(P_{\text{lx}}-PO_{\text{lx}}\right)}^{2}+{\left(P_{ly}-PO_{ly}\right)}^{2}\right]},\sqrt{{\left(P_{\text{lx}}-PO_{\text{lx}}\right)}^{2}+{\left(P_{ly}-PO_{ly}\right)}^{2}}\leq d_{0}\\ 0,\sqrt{{\left(P_{\text{lx}}-PO_{\text{lx}}\right)}^{2}+{\left(P_{ly}-PO_{ly}\right)}^{2}}>d_{0} \end{array}\right.$$

(6) $$U_{r}=\left\{\begin{array}{l} e^{-\eta \left[{\left(P_{rx}-PO_{rx}\right)}^{2}+{\left(P_{ry}-PO_{ry}\right)}^{2}\right]},\sqrt{{\left(P_{rx}-PO_{rx}\right)}^{2}+{\left(P_{ry}-PO_{ry}\right)}^{2}}\leq d_{0}\\ 0,\sqrt{{\left(P_{rx}-PO_{rx}\right)}^{2}+{\left(P_{ry}-PO_{ry}\right)}^{2}}>d_{0} \end{array}\right.$$

where $d_{0}$ is the maximum influence length of each obstacle, and η is a positive scalar. Afterward, the repulsive forces can be calculated by the gradient descent method as given in Equation 7.


(7) $$\left\{ \begin{array}{c} F_{\text{lrep}}=-\nabla U_{l}\\ F_{\text{rrep}}=-\nabla U_{r} \end{array}\right.$$

**Figure 4:**
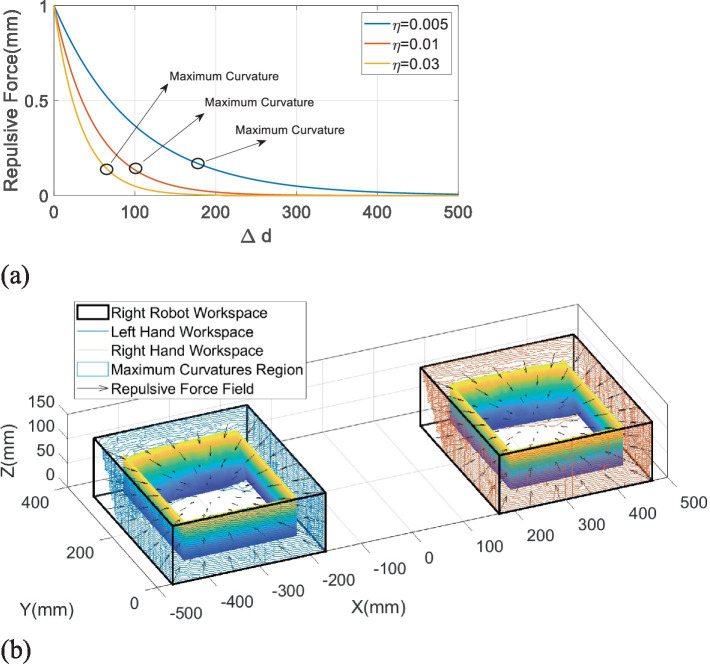
Maximum curvature dots of the repulsive potential field. (a) The maximum curvature dots of the different repulsive curves. (b) The repulsive potential field.

In the context of rehabilitation training, $d_{0}$ is generally fixed, while the parameter η should be customized to individual subjects based on their control ability of muscular strength. A big η generates an extensive but flat repulsive potential field, which limits interference range for a freedom movement and affects activities of limbs. However, a small η will form a narrow but steep repulsive potential field, which may reduce the compliance of the movement. As a consequence, it is essential to explore the most appropriate η that leads the trajectories of the handles stabilizing in a certain area. We assume that each repulsive force field consists of numerous repulsive force lines, as shown in Figure 4a. It is observed that the maximum curvatures of the lines, as well as the inflection points, are capable of balancing the repulsive force gradient and its range of influence. Hence, it needs to quantify the maximum curvature regions, which are described by colored surfaces, as shown in Figure 4b.

In response to this problem, an iterative learning method is used to hunt for the optimal η round by round, which can be described as given in Equation 8.


(8) $$\eta_{k+1}=\eta_{k}+\delta \Delta \eta$$

where δ is the learning rate, and subscript k means the kth positive scalar.

Denoting dl|Aη=ηk=Plx−iOPlx2+Ply−iOPly2 and dr|Aη=ηk=Prx−iOPrx2+Pry−iOPry2, the maximum curvature of the ith line for each side can be calculated as given in Equation 9.


(9) {Cilmax|Aη=ηk=maxdl|Aη=ηk≤d0|Aη=ηkFl’’1+Fl’22/3Cirmax|Aη=ηk=maxdr|Aη=ηk≤d0|Aη=ηkFr’’1+Fr’22/3

The corresponding plane coordinates of the maximum curvatures can be expressed as AiCPl=AiCPlx,iCPly for the left side and AiCPr=AiCPrx,iCPry for the right side. Then, the distances between the maximum curvature points and the boundary points are obtained as follows:


(10) {dCl|Aη=ηk=AiCPlx−iOPlx2+AiCPly−iOPly2dCr|Aη=ηk=AiCPrx−iOPrx2+AiCPry−iOPry2

The subject who intends to train is asked to move the handles along the workspace boundary, and sampling distances dl|Aη=ηk,dr|Aη=ηk will be recorded to compare with distances given in Equation 10 as follows:


(11) 1n∑i=1n||dClAη=ηk−dilAη=ηk||<ε

where ε is a predefined deviation threshold. If the inequality in Equation 11 is not satisfied, $\eta_{k}$ will not be used; then, the iteration continues (k→k+1); otherwise, the iteration stops, which means $\eta_{k}$ is the optimal scalar.

### 2.3 Training strategy and performance indicators

In mirror symmetry rehabilitation, reaching-task training is commonly implemented. To precisely evaluate the training performance, one clinical study established a linear regression model based on hundreds of stroke patients’ behavior information in Fugl-Meyer Assessment (FMA) scales. It emphasized three key indicators, including peak speed, smoothness, and duration (Bosecker et al., 2010). The peak speed represented the maximum velocity in one reaching training round, as defined in Equation 12. The smoothness signified the ratio of mean to peak speed, as denoted in Equation 13. The duration is the completion time of one target-to-target task, as described in Equation 14.


(12) $$P_{n}=\max_{1\leq i\leq n}\left\{\frac{\Delta d_{i}}{\Delta t_{i}}\right\}$$

(13) $$S_{n}=\frac{\sum_{i=1}^{m}\Delta d_{i}}{\sum_{i=1}^{m}\Delta t_{i}\cdot \max_{1\leq i\leq m}\left\{\frac{\Delta d_{i}}{\Delta t_{i}}\right\}}$$

(14) $$D_{n}=\sum_{i=1}^{m}\Delta t_{i}$$

where the subscript n means the nth raining round, and the parameter m represents the sample number in one round. $\Delta d_{i}$ is the displacement deviation between two contiguous samples, denoted as follows: $\Delta d_{i}=\sqrt{{\left(Pi_{x}-\text{Pi}-1_{x}\right)}^{2}+{\left(Pi_{y}-\text{Pi}-1_{y}\right)}^{2}}$. $\Delta t_{i}$ is the corresponding time deviation.

To scientifically define standard performance indicators, Fitts’s law was involved to determine desired duration at first (Fitts and Peterson, 1964), as given in Equation 15.


(15) $$D_{d}=a+b\cdot \log_{2}\left(\frac{L}{R}+1\right)$$

where R denotes the radius of the targets, and L represents the distance between any two targets. The parameters a and b are constant values, which are commonly set according to clinical training requirements.

Because desired peak speed and smoothness are both dependent on velocity, it is significant to define an appropriate trajectory between the two targets. There is clear evidence that the minimum jerk principle is able to characterize the reaching trajectory of upper limbs, which can be expressed as given in Equation 16 (Flash and Hogan, 1985).


(16) $$q\left(t\right)=L\left(10\tau^{3}-15\tau^{4}+6\tau^{5}\right)$$

where the parameter $\tau =t/D_{d}$. Then, the first-order derivative of Equation 16 can be acquired as given in Equation 17.


(17) $$v\left(t\right)=\frac{L}{D_{d}}\left(30\tau^{2}-60\tau^{3}+30\tau^{4}\right)$$

Furthermore, the parameter $P_{d}$ can be calculated as given in Equation 18.


(18) $$P_{d}=\max_{0\leq t\leq D_{d}}\left\{v\left(t\right)\right\}$$

Finally, the desired smoothness can be written as given in Equation 19.


(19) $$S_{d}=\frac{\int_{0}^{D_{d}}v\left(t\right)dt}{D_{d}P_{d}}$$

### 2.4 Control system design

**Figure 5:**
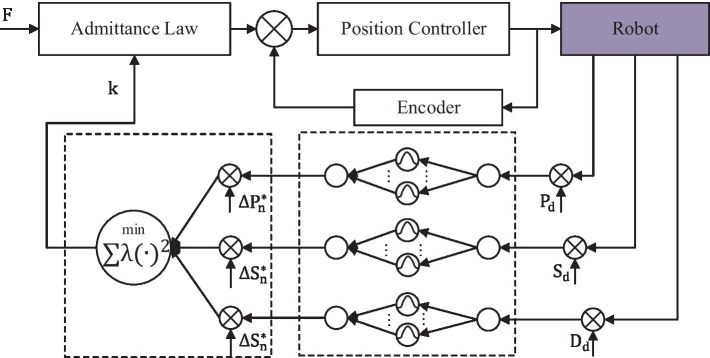
Overall control architecture of the robotic system.

Based on the above concepts, it is obvious that the smaller the gap between the desired and the measured performance indicators, the better the training effectiveness. For this purpose, the difficulty of training should be subject-specific. We assume that there exists the nth difficulty level $k_{n}\in {\mathbb{R}}^{+}$ such that the nth comprehensive performance error is minimum in each indicator’s limited range of variation, as given in Equation 20.


(20) $$\exists k_{n}\in {\mathbb{R}}^{+},s.t.\min\left\{\Delta P_{n},\Delta S_{n},\Delta D_{n}\right\}$$

(21) $$\left\{\begin{array}{l} \Delta P_{n}\in \left[-P_{thr},P_{thr}\right]\\ \Delta S_{n}\in \left[-S_{thr},S_{thr}\right]\\ \Delta D_{n}\in \left[-D_{thr},D_{thr}\right] \end{array}\right.$$

where $\Delta P_{n}=P_{d}-P_{n}$, $\Delta S_{n}=S_{d}-S_{n}$ and $\Delta D_{n}=D_{d}-D_{n}$ are the performance indicator errors, as given in Equation 21. $P_{thr}$, $S_{thr}$, and $D_{thr}$ are the threshold values of the corresponding variations. To make the thresholds appropriate, a physiotherapist is involved to give basic references at first. Then, they are further adjusted according to the feedbacks of the subjects after a series of previous experiments. Therefore, the mapping from the human functional ability to the robot resistance level and the multi-objective optimization should be considered. Based on this, the RBFNN method is employed to obtain the optimal difficulty level, as shown in Figure 5.

It assumed that $x=\left[\Delta P_{n},\Delta S_{n},\Delta D_{n}\right]$, the input is the single performance indicator error $x_{i}\in {ℜ}^{n}$. The output of the network is the difficulty level, which is the scalar function of the input indicator as given in Equation 22.


(22) $$f_{i}\left(x_{i}\right)=\sum_{j=1}^{N}\omega_{j}\rho \left(\left|\left|x_{i}-\mu_{j}\right|\right|\right),i=1,2,3$$

where N is the number of the nodes in the hidden layer, which is set at 20. $\mu_{j}$ is the center field, and $\omega_{j}$ is the jth weight. The radial basis function is defined as Gaussian form as given in Equation 23.


(23) $$\rho \left(\left|\left|x-c_{j}\right|\right|\right)=e^{\frac{-{\left||x_{i}-\mu_{j}|\right|}^{2}}{2\sigma^{2}}}$$

where σ is the standard deviation of the function. The RBF networks are trained by indicators, and the difficulty levels are sampled from previous experiments. Specifically, the chosen $\mu_{j}$ is the k-means clustering, and the σ can be obtained as given in Equation 24.


(24) $$\sigma =\frac{\mu_{\text{max}}}{\sqrt{2N}}$$

The least squares function is used to calculate the weights between the hidden layer and the output layer as given in Equation 25.


(25) $$\omega =e^{{\left||x_{i}-\mu_{j}|\right|}^{2}\cdot \frac{N}{\mu_{\text{max}}^{2}}}$$

The technique for order of preference by similarity to ideal solution (TOPSIS) method is employed for multi-objective optimization. It is assumed that the desired minimum errors are $\Delta P_{n}^{*}$, $\Delta S_{n}^{*}$, and $\Delta D_{n}^{*}$. The model can be written as in Equation 26.


(26) $${\tilde{k}}_{n}=\sum_{i=1}^{3}\lambda_{i}{\left(x_{i}-x_{i}^{*}\right)}^{2}=\sum_{i=1}^{3}\lambda_{i}{\left[f_{i}^{-1}\left(x_{i}\right)-x_{i}^{*}\right]}^{2}$$

where $x^{*}=\left[\Delta P_{n}^{*},\Delta S_{n}^{*},\Delta D_{n}^{*}\right]$, and $\lambda_{i}$ is the ith weight of the ith objective function. $f_{i}^{-1}\left(x_{i}\right)$ is the ith inverse function of the ith performance indicator error.

The admittance law module makes the device operate with specific inertia, specific damping, and unfixed stiffness by measuring and controlling the force from two force sensors. These parameters are equal on the X-axis and the Y-axis. The admittance equation is written as given in Equation 27.


(27) $$\left\{\begin{array}{l} F_{l}=m_{l}{\ddot{q}}_{l}+b_{l}{\dot{q}}_{l}+k_{l}q_{l}\\ F_{r}=m_{r}{\ddot{q}}_{r}+b_{r}{\dot{q}}_{r}+k_{r}q_{r} \end{array}\right.$$

where $F_{l}={\left[F_{l}^{x},F_{l}^{y}\right]}^{T}$ denotes the measured interactive force vector on the left handle along the X-axis and the Y-axis, and $F_{r}={\left[F_{r}^{x},F_{r}^{y}\right]}^{T}$ corresponds to the right handle. The parameters $\left(m_{l},m_{r}\right)$, $\left(b_{l},b_{r}\right)$, and $\left(k_{l},k_{r}\right)$ represent the predefined robotic handle’s mass, damping, and stiffness factors depending on specific tasks, respectively. Setting the trajectories caused by the interactive forces, the admittance law can be simplified into Equation 28 as a linear spring, where the acceleration and the velocity are ignored (Ott et al., 2015).


(28) $$\left\{ \begin{array}{l} F_{l}=k_{l}q_{l}\\ F_{r}=k_{r}q_{r} \end{array}\right.$$

Combined with the repulsive potential function and the training difficulty level, Equation 28 can be modified as given in Equation 29.


(29) $$\left\{\begin{array}{l} F_{l}={\tilde{k}}_{l}q_{l}+F_{\text{lrep}}\\ F_{r}={\tilde{k}}_{r}q_{r}+F_{\text{rrep}} \end{array}\right.$$

## 3 Experimental results

### 3.1 Experimental protocol

The experiments were conducted with the end-effector-based bilateral robot to validate the feasibility of the developed safety metrics. Two healthy subjects (two male participants: age 29.00 ± 4.24 years, height 1765.00 ± 21.21 mm, and weight 83.00 ± 9.90 kg) volunteered to participate in this study. The study was approved by the Southern University of Science and Technology, Human Participants Ethics Committee (20190004), and consent was obtained from the participant.

To test the performance of the proposed safety strategy, the experiments were divided into two blocks. The first experiment was conducted to search for the most appropriate η values of each subject. The subjects were required to execute a reaching-task training between two pre-set points for the first 20 rounds. Then, the subjects needed to move the left handle along the workspace boundary in an anticlockwise direction and synchronously move the right side in an anticlockwise direction (mirror symmetry training mode) during the next 20 rounds. In this context, the positions of the targets were set at [−400.00 mm, 200.00 mm] and [−230.00 mm, 50.00 mm] for the left side and [400.00 mm, 200.00 mm] and [230.00 mm, 50.00 mm] for the right side. The admittance parameters were fixed as ${\tilde{k}}_{l}={\tilde{k}}_{r}=0.08$, which could make subjects’ movements more compliant. The starting η value was set at 400. The deviation Δη was set at 30. Due to the large learning rate δ causing large η that limits interference range for a freedom movement but the small learning rate δ extending the optimization time, the learning rate δ was finally set at 0.5 to combine the rate of convergence of η and the training efficiency after proceeding numbers of preliminary experiments and seeking advice from a physical therapist.

In the second experiment, the performance-based robotic assistance strategy was added to validate whether it is effective to approximate the training tasks, including four difficulty levels with 40 rounds of training (each for 10 rounds). The positions of the targets were set as in the first experiment. According to a series of preliminary training tests, the parameters a and b in Fitts’s law were set both at 1. Combined with Equations 15 to 19, the desired performance indicators can be worked out, as shown in Table 1. Due to all the subjects being healthy individuals, the initial ${\tilde{k}}_{l}$ and ${\tilde{k}}_{r}$ were set at 0.1, and the range of admittance values was limited in [0, 0.12]. The desired minimum errors were set as $\Delta P_{n}^{*}$=15 mm/s, $\Delta S_{n}^{*}$=0.15, and $\Delta D_{n}^{*}$=0.5 s. After acquiring 1,200 groups of the performance indicators of each subject and corresponding admittance parameter, the customized values can be obtained (as shown in Table 1 for the first subject and Table 2 for the second subject).

**Table 1:** Definition of the desired performance indicators and the training results of the admittance parameters for the first subject.

Round (No.)	*P_d_* (mm/s)	*S_d_*	*D_d_* (s)	*k_l_*	*k_r_*
0–10	94.46	0.53	4.5	0.0159	0.0166
10–20	121.45	0.53	4.5	0.0231	0.0241
20–30	170.05	0.53	4.5	0.0492	0.0498
30–40	283.39	0.53	4.5	0.0764	0.0791

**Table 2:** Definition of the desired performance indicators and the training results of the admittance parameters for the second subject.

Round (No.)	*P_d_* (mm/s)	*S_d_*	*D_d_* (s)	*k_l_*	*k_r_*
0–10	94.46	0.53	4.5	0.0232	0.0245
10–20	121.45	0.53	4.5	0.0312	0.0337
20–30	170.05	0.53	4.5	0.0582	0.0611
30–40	283.39	0.53	4.5	0.0776	0.0808

### 3.2 Experimental results

**Figure 6:**
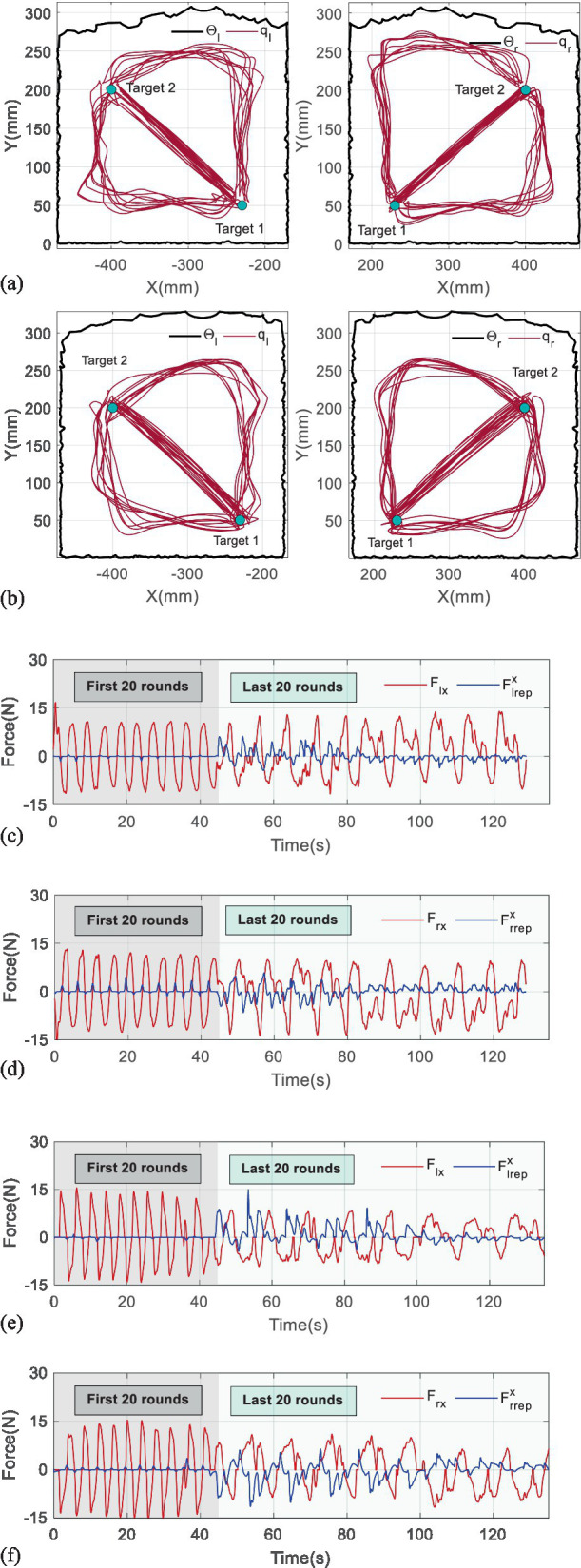
Results of the first experiment. (a) The trajectories being generated by the first subject. (b) The trajectories being generated by the second subject. (c) The interactive and repulsive forces performed by the first subject on X-axis. (d) The interactive and repulsive forces performed by the first subject on Y-axis. (e) The interactive and repulsive forces performed by the second subject on X-axis. (f) The interactive and repulsive forces performed by the second subject on Y-axis.

Figure 6 reports the results of the first experiment, where Figures 6a,b the trajectories, respectively, being generated by two subjects. Figures 6c,d display their interactive forces (red lines) and repulsive forces (blue lines) on X-axis, while Figures 6e,f correspond to the forces on Y-axis.

It is found that the trajectories in the first 20th training rounds consist of straight lines, which reflect that the subjects adapted well to the mirror symmetry training, and repulsive forces appear only if the handles approach the targets. Owing to the small η, the repulsive forces exponentially increase when the handles are close to the boundaries of the workspaces at the beginning of the last 40 rounds. However, the repulsive forces gradually reduce with the η continuously modulating in approximately 8–10 rounds, which verifies that the trajectories can converge to maximum curvature points.

**Figure 7:**
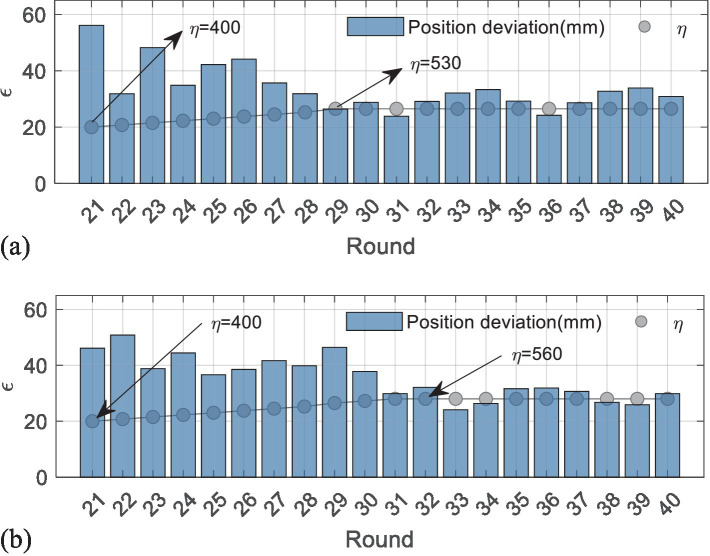
Iterative process of the first experiment. (a) The results of the first subject. (b) The results of the second subject.

In detail, the iterative processes of the η are given in Figure 7, where the blue bars represent the position deviations, and the gray dots are corresponding η. To make it clearer to analyze, the values of η in Figure 7 are multiplied by 0.02. The data are recorded from the 21th round and provided in Table 1. The results in Figure 7a show that the position deviations from the 21th rounds to the 29th rounds are far more beyond the predefined ε=30 mm, so the η increases from 400 to 530. In contrast, the η holds when the position deviation is below 30 mm, which means iteration stops, and η=530 ought to be the optimal scalar for the first subject. Although some position deviations, such as in the 33–35th round, are not completely smaller than ε=30 mm, their difference values are in few millimeters, which can be assumed to be effective. Similarly, the most appropriate η for the second subject can be determined as 560 in Figure 7b.

**Figure 8:**
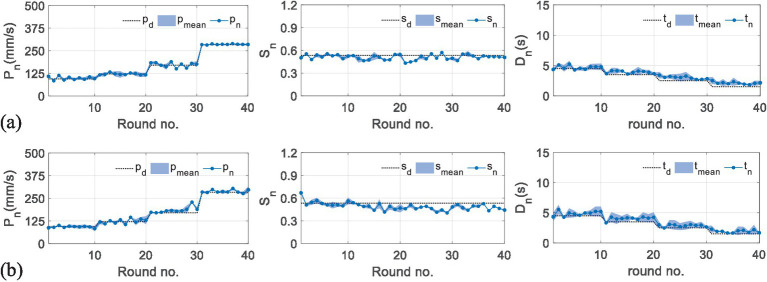
Results of measured performance indicators applied by the subjects in the second experiment. (a) The results of the first subject. (b) The results of the second subject.

**Figure 9:**
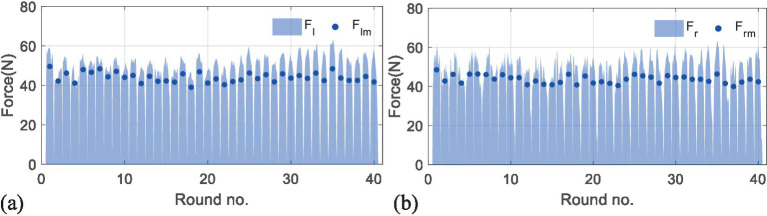
Results of measured interactive forces in the second experiment. Figure 9 (a) represents the mean interactive forces performed by the left hands of both subjects. The light blue shadows represent measured forces, and the blue dots represent corresponding mean values. Figure 9 (b) shows the results of the right side.

Figures 8,9 present the results of the second experiment, where Figure 8 shows the measured performance indicators applied by the subjects in the second experiment. The black and gray imaginary lines represent different desired performance indicators. Figure 8a uses blue lines to represent the measured performance indicators of the first subject and applies light blue shadows to describe the standard deviations. Figure 8b represents the results performed by the second subject. Figure 9 gives measured interactive forces in the second experiment.

**Figure 10:**
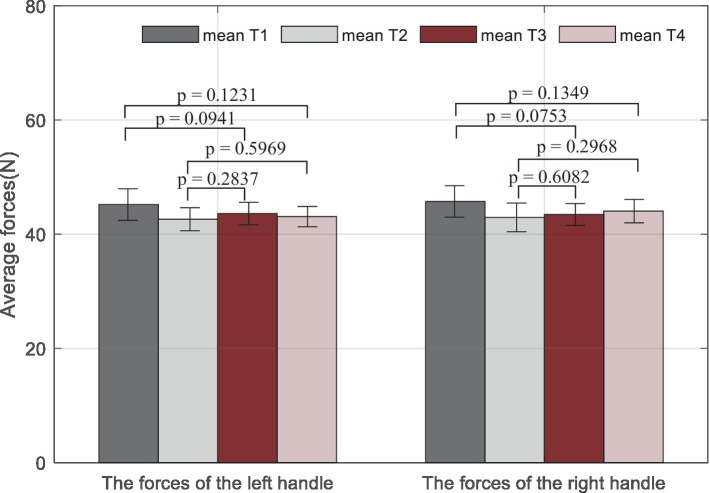
Statistical analysis results of average measured forces on the left handle and the right handle during the training. Mean Ti (i = 1,2,…, 4) represents the mean force performed by both subjects during the ith trial.

A statistical analysis with a paired *t*-test is used for comparisons among the trials. It is denoted that four training tasks correspond to T1, T2, T3, and T4. The results given in Figure 10 show that the *p*-values of the *t*-test are all larger than 0.05, which means there are no significant differences represented between any two tasks, either for the left hand or the right side.

Overall, it can be seen that the measured indicators approximate the desired values. For more specific information, in a total of 40 rounds, the values of the root-mean-square error (RMSE) for the first subject are 26.24 mm/s, 0.06, and 0.02 s, and 22.41 mm/s, 0.04, and 0.12 s for the second subject, which shows the feasibility of the RBFNN-based method. The average forces in Figure 8 vary steadily during the whole training, which implies that the training difficulty levels fit the subjects well, and the training effectiveness tends to be positive.

## 4 Discussion

Robot-assisted upper-limb training plays an important role in reducing the burden of labor and improving the training efficiency. To guarantee the safety of the robotic system and provide appropriate assistance, previous studies preferred to define uniformed workspace and rigid motion restraint as the safe metrics. However, these strategies ignored the human specificity and motion smoothness. To improve the accuracy of performance evaluation, some studies used multi-performance-based control methods to synthetically adjust parameters of the robotic system (Krebs et al., 2003; Papaleo et al., 2013), while few studies focus on exploring subject-specific training methods to maximize subject participation.

The developed integrated framework can benefit robot-assisted rehabilitation training in three aspects. First, this study developed subject-specific workspaces based on human kinematic information and the robot characteristic to ensure the training safety. Second, the proposed iterative learning-based repulsive force field is capable of providing optimal motion constraints, which can reduce the risk of secondary injury and avoid unbalance between movement freedom and compliance. Finally, the designed robotic assistance strategy introduces three performance measures that are closely linked to clinical scales to improve the evaluation accuracy of training, and a learning method combined with the repulsive force field is developed to obtain customized control parameters for various that can approximate any training requirements.

Experiments on healthy subjects are enrolled to validate the safety and feasibility of the proposed framework. The results show that the framework is capable of guaranteeing safe and natural movements and providing different subject-specific parameters for individuals to conduct various training tasks. Furthermore, the results shown in Figure 8 in this article present better rapidity than the results in our previous study (Miao et al., 2023). The fuzzy-based methods need several iteration times to lock appropriate robotic assistance, while the RBFNN-based control structure can skip the convergence procedure, which can increase training efficiency.

However, there are still some limitations to this study. First, the training tasks are defined only in a two-dimensional space, while most activities of daily living belong to the category of three-dimensional space. Second, the learning strategy relies on long time for offline training. Third, the experiments only include healthy individuals. However, it should be noted that the human kinematic upper-limb model can be achieved according to the FMA scales, and all involved control parameters are able to be trained or further optimized for various groups of subjects; hence, the system is also applicable to patients.

## 5 Conclusion

This study proposes an integrated framework for robot-assisted upper-limb training, which not only includes human kinematic-based compliant motion constraints for safe interactive training but also develops a performance-based adaptive control strategy to provide appropriate robotic assistance. Experimental results demonstrated that the proposed framework can avoid unsafe motion and prompt the acquisition of appropriate subject-specific parameters. Future studies will consider the optimization of the proposed framework with advanced algorithms, as well as its clinical application with a larger sample size of patients.

## Data Availability

The original contributions presented in the study are included in the article/supplementary material, further inquiries can be directed to the corresponding authors.
